# Research progress on sucrose accumulation characteristics and their molecular regulatory networks in the fruits of fruit trees

**DOI:** 10.3389/fpls.2026.1875616

**Published:** 2026-07-03

**Authors:** Yushuang Geng, Yujia Luo, Zhi Luo, Ping Xiong, Fenfen Yan

**Affiliations:** 1 Southern Xinjiang Distinctive Forestry & Pomology Technology Innovation Center, Tarim University, Xinjiang Production and Construction Corps, Alar, China; 2 College of Horticulture and Forestry, The National and Local Joint Engineering Laboratory of High Efficiency and Superior-Quality Cultivation and Fruit Deep Processing Technology of Characteristic Fruit Trees, Tarim University, Alar, China; 3 College of Life Science and Technology, The National and Local Joint Engineering Laboratory of High Efficiency and Superior-Quality Cultivation and Fruit Deep Processing Technology of Characteristic Fruit Trees, Tarim University, Alar, China

**Keywords:** accumulation traits, fruit tree, genes, molecular regulatory networks, sucrose

## Abstract

Sucrose is a key determinant of fruit sweetness and flavor quality. However, sucrose accumulation varies greatly among fruit tree species, and the molecular regulatory mechanisms and gene networks underlying this variation have not been systematically analyzed, which limits deeper insights into fruit quality improvement. Here, based on the sucrose accumulation characteristics of mature fruits, we classify major fruit trees into three types: sucrose-accumulating, intermediate-converting, and sucrose-hydrolyzing, and clarify the differences in sucrose metabolism and accumulation among them. We further integrate QTL mapping and multi-omics analyses to systematically catalog sucrose regulatory genes in fruit trees. Focusing on the pathways of synthesis, transport, and storage, we analyze the molecular regulatory network of sucrose accumulation and reveal the underlying molecular mechanisms in different fruit trees. We also clarify the regulatory effects of endogenous hormones and environmental factors on sucrose metabolism. This review aims to advance the theoretical framework of molecular regulation of sugar accumulation in fruit trees and to provide theoretical references and technical support for flavor quality improvement and precision breeding for sugar content in fruit.

## 1 Introduction

Sucrose is the main form of photoassimilates for long-distance transport in higher plants, and it also serves as a core determinant of sweet taste perception and flavor quality in fruits. Unlike glucose and fructose, sucrose not only directly contributes to sweetness but also acts as an energy carrier and signal molecule, thereby regulating the activities of downstream metabolic enzymes and transporters and dominating overall sugar accumulation in fruits (Su et al., 2022; Li JH. et al., 2025). In recent years, forward and reverse genetic strategies have been successfully applied in tree species with relatively complete research systems, such as apple and citrus, leading to the identification of multiple sucrose metabolism-related genes (e.g., MdISA3 in apple (Wang, 2022) and PpTST1 in peach (Shi, 2021)). Moreover, numerous differentially expressed genes and regulatory modules have been identified (Zhu et al., 2024; Liu SC. et al., 2025). Nevertheless, common challenges in fruit trees, such as high genomic heterozygosity and long generation cycles, result in generally low resolution of existing QTL mapping intervals, hindering the precise identification of key candidate genes within these intervals (Sabety et al., 2024). Meanwhile, because functional validation systems are still immature, the functional characterization of many candidate genes continues to rely on heterologous systems (e.g., tomato and Arabidopsis), with limited direct evidence from homologous transformation; this slows the systematic elucidation of the regulatory network for sucrose accumulation (Campa et al., 2024). These dilemmas are particularly prominent in less-studied fruit trees with relatively weak research foundations.

To address the gaps in our understanding—namely, the unclear mechanisms underlying interspecific differences and the lack of systematic summaries of regulatory networks—we systematically integrate recent findings in fruit tree sucrose metabolism. Based on the differences in sucrose accumulation in mature fruits, we classify major fruit trees into sucrose-accumulating, intermediate-converting, and sucrose-hydrolyzing types and summarize their differential characteristics. We further evaluate the applications and technical limitations of multi-omics technologies in the discovery of sucrose-related genes. Focusing on the three core processes of synthesis, transport, and storage, we integrate and analyze the interaction networks among key genes and regulatory elements. Finally, we outline future research priorities and potential breakthroughs, aiming to advance the theoretical framework for the formation of sugar-related quality traits in fruit and to provide scientific support for precise flavor improvement and molecular breeding.

## 2 Characteristics and types of sucrose accumulation in fruit tree fruits

### 2.1 Comparison of sucrose accumulation in fruits of different fruit trees

Sucrose is a non-reducing disaccharide formed by glucose and fructose linked via an α, β-1, 2 glycosidic bond, and serves as the major form for long-distance transport of photoassimilates in higher plants (Su et al., 2021). In most fruit trees, sucrose content is low in the early stage of fruit development, and soluble sugars mainly exist in the form of hexoses (glucose and fructose). The significant accumulation of sucrose usually occurs at the late developmental or mature stage (Lu et al., 2022). There are significant differences in the proportion of sucrose accounting for total soluble sugars in fruits at the late developmental stage (i.e., maturity stage) among different fruit tree species. For example, the proportion of sucrose in total soluble sugars of jujube fruit can reach 50%-77% (Zhao et al., 2016; Deng, 2019); the sucrose proportion in peach fruit is approximately 67% (Xu, 2021); Citrus ranges from 41.54% to 64.60% (Lin et al., 2021). Thus, sugar accumulation in these fruits is dominated by sucrose, with a relatively high proportion of sucrose in soluble sugars. The proportion of sucrose in soluble sugars of mature apple fruits ranges from 11% to 40% (Zhao et al., 2025), and that of pear is 4%–12.9% (Song et al., 2012; Zhang S. et al., 2025). The value of strawberry is about 37.85% (Su et al., 2011), while sucrose only accounts for 1.61% of total sugars in mature grape fruits (L.A. and Carroll, 1973). Thus, sugar accumulation in these fruits is not dominated by sucrose, with a relatively low proportion of sucrose in soluble sugars.

### 2.2 Classification and variation of sucrose accumulation patterns in fruit tree fruits

Based on the differences in sucrose accumulation in mature fruits, major fruit trees can be divided into three types: Sucrose-accumulation type: With high sucrose content (usually accounting for more than 50% of total soluble sugars), sucrose accumulates continuously during the middle and late stages of fruit development and is directly stored in vacuoles, such as jujube and citrus. Intermediate-converting type: It has a moderate sucrose content (approximately 15%–40%). Sucrose is hydrolyzed in the early stage of fruit development, then resynthesized via the sorbitol pathway at the mature stage, and coexists with hexoses, such as apple and pear. Sucrose-hydrolyzing type: It has an extremely low sucrose content (usually less than 10%). After sucrose is unloaded from the transport pathway, it is completely hydrolyzed into hexoses, and the fruit is mainly composed of glucose and fructose, such as grape and strawberry.

#### 2.2.1 Sucrose-accumulating type

**Figure 1:**
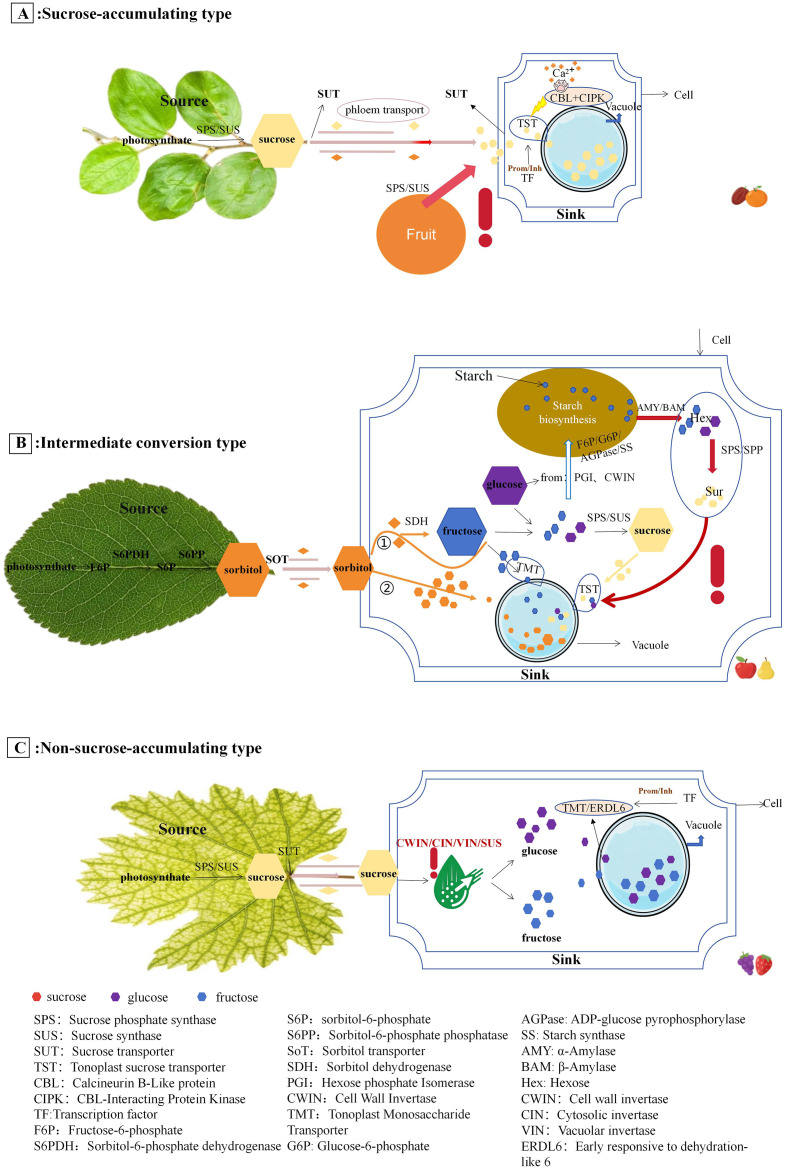
Schematic diagram of three patterns of sucrose unloading and accumulation in fruit tree fruits. **(A)** Schematic diagram of metabolism and transport in sucrose-accumulating type; **(B)** Schematic diagram of metabolism and transport in intermediate-converting type; **(C)** Schematic diagram of metabolism and transport in sucrose-hydrolyzing type.

Sucrose-accumulation type is typically represented by jujube (Rhamnaceae) and citrus (Rutaceae). At fruit maturity, sucrose accounts for over 50% of soluble sugars (jujube can exceed 70%) (Xue et al., 2024; Wu et al., 2025). This is because after sucrose enters flesh cells, it is directly stored in vacuoles; the activity of the hydrolysis pathway is low while that of the synthesis pathway is high, thereby achieving efficient net accumulation of sucrose. In research on jujube, reducing sugar content is high at the early stage of fruit development. The activities of invertase (AI, NI) and sucrose synthase in the cleavage direction (SS-CD) remain relatively high, which can rapidly decompose sucrose into glucose and fructose. Consequently, sucrose content exhibits an extremely significant negative correlation with the activities of catabolic enzymes (Guo et al., 2019). In the middle and late stages of fruit development, invertase activity decreases, while the activities of sucrose synthesis-related enzymes (SS-SD, SPS) increase significantly. Sucrose begins to accumulate in large quantities and shows a significant positive correlation with the activities of synthetic enzymes, forming a pattern of sucrose accumulation characterized by enhanced synthesis and weakened decomposition (Guo et al., 2019). The sucrose content of ‘Shucui jujube’ at maturity shows an extremely significant positive correlation with the expression of synthetic genes *ZjSS3*, *ZjSPS1* and *ZjSPS2*, and a significant negative correlation with the expression of the catabolic gene *ZjvINV2*. This suggests that the above changes in enzyme activity result from the coordinated regulation of gene expression (Wang et al., 2022). The difference in fruit sweetness between cultivated jujube and wild sour jujube essentially depends on the sucrose content: the transporter *ZjSWEET15* is highly expressed in cultivated jujube and promotes sucrose accumulation, whereas sucrose synthase *ZjSUS2* exhibits higher cleavage activity in wild sour jujube and inhibits sucrose storage. The coordination of the two forms a high-sucrose pattern characterized by enhanced transport and weakened decomposition (Zhang Z. et al., 2025). In recent years, multiple sucrose phosphate synthase genes *CsSPS1-CsSPS4* have been identified in citrus, whose expression is positively correlated with fruit sugar accumulation (Lu W. et al., 2024). Using transcriptome analysis of citrus hybrid populations, researchers identified the transcription factor CitERF36, and its expression level is significantly positively correlated with fruit total soluble solids (TSS) and sucrose content. Further studies have shown that *CitERF36* promotes net sucrose accumulation by coordinately regulating both sucrose transport and catabolism pathways: on the one hand, it directly activates the expression of the plasma membrane sucrose transporter gene *CitSUT2*, enhancing the transport of sucrose into flesh cells; on the other hand, it represses the transcription of the vacuolar acid invertase gene *CitvINV3*, reducing the degradation and consumption of sucrose in vacuoles. This coordinated regulation of enhanced transport and reduced degradation represents the molecular regulatory hallmark of efficient sucrose accumulation in citrus fruits (Pang et al., 2026). In addition, multiple upstream signaling pathways are involved in regulating sucrose transporters: the transcription factors *CsMYBS3* and *CsbHLH122* interact to form a transcriptional complex, which synergistically enhances the promoter transcriptional activity of *CsSUT2* and rapidly improves sucrose transport efficiency during the late stage of fruit ripening (Zhai et al., 2025). Studies have found that the transcription factor *CitSAR*, as a master regulator, can simultaneously activate the sucrose phosphate synthase gene *CitSPS4* to promote sucrose accumulation, and accelerate the catabolic balance of citric acid, thereby affecting fruit flavor and quality (Liu SC. et al., 2025). In summary, both jujube and citrus follow this regulatory pattern, and the core characteristics of their sucrose-accumulating type fruits can be summarized as enhanced synthesis, weakened degradation, and efficient transport. It is worth noting that the exploration of upstream transcription factors in citrus (such as *CitERF36* and *CitSAR*) has been relatively systematic, forming a molecular model of multi-level coordinated regulation. In jujube, although there are precedents of upstream transcription factors (such as *ZjABF1*) regulating transporters, the overall regulatory network remains to be further constructed and improved (**Figure 1A**).

#### 2.2.2 Intermediate-converting type

Intermediate-converting type is typically represented by apple and pear (Rosaceae), with sucrose accounting for approximately 30% of soluble sugars at fruit maturity. This is due to the “breakdown-then-synthesis” pattern of sucrose: in the early stage, photosynthates are transported to fruits in the form of sorbitol; after degradation, part of the carbon flux is temporarily stored as starch (Yuan et al., 2023). When the fruit enters the maturation stage, starch is degraded into hexoses, which are then resynthesized into sucrose via SPS, ultimately forming a pattern where sucrose and hexoses coexist (Yi et al., 2023; Yuan et al., 2023). In apple, the cell wall invertase gene *MdCWINV1* is a key gene regulating sucrose breakdown and sugar accumulation in apple fruit. Its encoded protein catalyzes the hydrolysis of sucrose into hexoses, directly participating in sucrose hydrolysis metabolism in fruit. Studies have shown that the transcription factor *MdWRKY20* can specifically bind to the promoter region of *MdCWINV1* and regulate its gene expression. However, natural variations in the *MdCWINV1* promoter weaken the binding ability of *MdWRKY20*, repress its transcriptional expression, reduce cell wall invertase activity, decrease sucrose hydrolysis and consumption, and ultimately alter hexose accumulation and reduce overall sugar content in fruit. This indicate that the sucrose catabolism mediated by *MdCWINV1* is an important pathway that negatively regulates sugar accumulation in apple fruits (Wang et al., 2026). Using pear as the research material, researchers found that the transcription factor PbrbZIP15 targets and activates the glucose isomerase gene *PbrXylA1*. By enhancing the conversion efficiency from glucose to fructose, it coordinately promotes the accumulation of sucrose and total sugars, thereby providing a novel regulatory mechanism for sugar metabolism in intermediate-converting type fruits (Jia et al., 2023).

#### 2.2.3 Sucrose-hydrolyzing type

Sucrose-hydrolyzing type is represented by berries such as grape and strawberry. At the mature stage, glucose and fructose dominate the sugar composition, with sucrose accounting for approximately 10% (Sun et al., 2021; Li, 2023). This is because of the complete hydrolysis of sucrose into stored hexoses: at fruit maturity, the activity of acid invertase increases significantly, which completely hydrolyzes sucrose into hexoses, and these hexoses are then stored via the tonoplast sugar transporter TMT (Lu et al., 2024b; Liu J. et al., 2025). Grape fruits have weak sucrose synthesis capacity, but sucrose unloaded from the phloem is rapidly hydrolyzed into hexoses by invertase and sucrose synthase (Sun et al., 2021). Meanwhile, the transcription factors *VvERF105* and *VvNAC72* drive the expression of hexose transporter genes through temporal regulation, efficiently transporting the hydrolyzed hexoses into vacuoles for storage, thereby aggravating sucrose consumption (Lu et al., 2024a). Strawberry fruits also mainly accumulate hexoses. During fruit maturation, the activity of acid invertase (e.g., *FaCWINV1*) increases significantly, rapidly hydrolyzing sucrose into hexoses. In addition, the E3 ubiquitin ligase COP1 degrades key regulatory factors that promote sugar synthesis via the ubiquitination pathway, further restricting sucrose synthesis and consolidating the metabolic pattern of hexose accumulation (Bi et al., 2025) (**Figures 1B, C**).

## 3 Research on sucrose-related genes in fruit trees

### 3.1 Research on sucrose regulatory genes based on QTL mapping

Sucrose content is regulated by multiple genes and is a typical quantitative trait (Li et al., 2023; Zhai et al., 2025). QTL mapping enables the precise genetic locus dissection and interval anchoring of complex sugar traits controlled by polygenes, serving as a crucial approach to identify key genes governing sucrose content and elucidate the genetic basis in fruit trees (Fang and Liu, 2022; Jiang et al., 2025). In recent years, it has been widely applied in major fruit trees such as apple, peach, citrus and jujube. A number of genetic loci and core candidate genes closely associated with sucrose synthesis, transport and accumulation have been successively identified, laying an important theoretical foundation for clarifying the mechanism of sugar differentiation in different tree species and exploring key regulatory genes of sucrose.

Numerous QTLs and functional genes associated with sucrose accumulation have been successfully identified in sucrose-accumulating type fruit trees. Two major QTLs for sucrose accumulation have been identified in jujube, mapped to linkage groups LG8 and LG1, with phenotypic contribution rates of 28.72% and 23.37%, respectively (Chen, 2024); multiple regulatory loci controlling sucrose content have been screened in citrus populations, with the phenotypic variance explained ranging from 12% to 37%. Several sucrose QTLs are co-localized with quality traits such as fruit weight and acidity, revealing a synergistic genetic regulatory basis between sucrose accumulation and comprehensive fruit quality (Khefifi et al., 2022). For peach, another sucrose-accumulating type fruit tree, key candidate genes including *PpTST1* and *PpVPS51* were screened within the LG5 region using a high-density SNP genetic map. Among them, non-synonymous variations in the coding region of *PpTST1* can significantly regulate the level of sucrose accumulation in fruit (Shi, 2021). Overall, most of the core genes screened from the above QTL intervals are responsible for key processes such as sugar transport and vacuolar storage, which are highly consistent with the physiological characteristics of efficient accumulation and stable storage of sucrose in sucrose-accumulating type fruits. This further confirms the central regulatory role of such genes in the accumulation of sucrose in fruit trees.

In apple, the representative tree species of intermediate-converting type, seven QTLs associated with sucrose content were identified, and two key candidate genes with clarified functions were identified. Among them, the isoamylase gene *MdISA3*, located on LG15, could significantly reduce fruit sucrose content by 32% when silenced, while overexpression of its superior haplotype can effectively promote sucrose enrichment and accumulation (Wang, 2022); the cell wall invertase gene *MdCWINV1* is mapped to LG03. Natural variations in its promoter region alter the binding efficiency of the transcription factor *MdWRKY20*, thereby inhibiting the transcription of *MdCWINV1* and cell wall invertase activity. This ultimately leads to reduced fructose synthesis and changes in sugar composition (Wang et al., 2026). The functions of these two genes are markedly differentiated. *MdISA3* primarily mediates starch degradation, whereas *MdCWINV1* is responsible for sucrose hydrolysis. This functional divergence precisely matches the carbon metabolic characteristics of intermediate-converting type fruit trees, namely starch conversion, sucrose breakdown, and resynthesis, thereby further confirming the unique sugar transformation mechanism of this fruit tree type at the genetic and molecular levels.

For strawberry, a representative species of the sucrose-hydrolyzing type, preliminary genetic mapping of QTLs associated with sucrose content has been completed (Ürün et al., 2025). Although fine cloning and functional validation of the key genes have not yet been achieved, the current mapping results have laid a preliminary foundation for dissecting the genetic regulatory mechanisms of hexose-dominated sugar metabolism in fruits.

Although a large number of sucrose metabolism-related genetic loci and functional genes have been identified in all three types of sucrose-accumulating type fruit trees, fruit trees generally exhibit characteristics such as long growth cycles, high genomic heterozygosity, and complex genetic backgrounds. These factors severely restrict the fine mapping of sucrose-related QTLs and the mining of candidate genes. Currently, most QTLs for sucrose-related traits have large physical intervals, reaching 2–4 Mb in jujube, 2–12 Mb in citrus, and 1–7 Mb in apple (**Table 1**), resulting in limited mapping resolution. Furthermore, current studies have mostly focused on single-gene or single-pathway analyses, leaving the multi-gene synergistic regulatory network of sucrose metabolism largely unclear. Moreover, a systematic comparison of conserved metabolic mechanisms and species-specific differences among different fruit tree species and sucrose accumulation types is lacking, which hinders the in-depth elucidation of the common regulatory mechanisms underlying sucrose accumulation in fruit trees.

**Table 1:** Comparison of QTL mapping accuracy related to sugar content in different fruit trees.

Tree species	Type of mapping population	Size of mapping population(individual number)	QTL linkage group/mapping resolution (Mb)
Jujube	F_1_ hybrid population (Female parent ‘Yuhong’ × Male parent ‘Jiaocheng No. 5’) (Chen, 2024)	142	LG5(2. 19) LG8(3. 64) LG1(2. 03) LG4(2. 90) LG1(0. 00) LG12(3. 95) LG5(2. 19)
Citrus	F_1_ hybrid population (Female parent ‘Clementine’ × Male parent ‘Liuye Mandarin’) (Khefifi et al., 2022)	105	LG9(3. 40) LG2(11. 70) LG3(4. 30) LG9(2. 60) LG9(3. 30) LG9(2. 60) LG5(3. 10)
Strawberry	F_1_ hybrid population (*Fragaria × ananassa* ‘Fortuna’ × *Fragaria × ananassa* ‘Rubygem’) (Ürün et al., 2025)	164	Fvb7(3. 66) Fvb7-3(13. 02) Fvb7(3. 66) Fvb2-3(2. 41) Fvb2-3(13. 63)
Apple	2 F_1_ hybrid population (‘Honeycrisp’ × ‘Qinguan’) (Wang, 2022; Wang et al., 2026)	113	LG08(1. 80) LG15(2. 04) LG15(5. 34) LG15(0. 01) LG12(0. 55) LG15(1. 13) LG15(6. 63)
Peach	F_1_ hybrid population ‘Shahong’ × ‘Hongfurong’ (Liu SC. et al., 2025)	202	1(0. 05) 1(1. 50) 1(0. 37) 1(0. 43) 1(0. 48) 1(0. 67) 1(1. 33) 4(0. 05) 5(0. 37) 5(1. 81) 6(0. 24) 6(1. 05)

This table summarizes the basic experimental information of QTL mapping studies related to sugar quality in five horticultural fruit tree species, including the type of mapping population, population size, linkage group/chromosomal location of QTLs, and physical mapping resolution. All studies were based on artificially constructed F_1_ hybrid segregation populations. The physical intervals of QTLs varied significantly across different tree species and loci, providing a reference for the excavation of key genetic loci and functional gene validation associated with sugar accumulation in fruit trees.

### 3.2 Research on sucrose regulatory genes based on multi-omics

In addition to QTL mapping, multi-omics strategies such as transcriptome sequencing (RNA-seq) and metabolomics provide a high-throughput approach for screening candidate genes involved in sugar metabolism. Among these, transcriptomics and metabolomics feature mature technological systems and high data reproducibility, enabling direct correlation between gene expression and changes in sugar component phenotypes. They are currently the most widely used high-throughput approaches in research on sucrose regulation in fruit trees. Proteomics and epigenomics can complement and refine the sucrose molecular regulatory network from the levels of protein abundance, post-translational modifications, and epigenetic regulation, respectively. Genomics, spatial omics, and other related omics approaches are rarely applied in this direction. The development degree of various omics approaches in studying sucrose mechanisms in fruit trees exhibits significant imbalance, due to the primary-secondary division of labor among different omics in their research positioning, their varying technical difficulty, combined with the objective constraints of fruit tree materials and experimental systems.

In sucrose-accumulating type fruit trees, key genes regulating sucrose transport and synthesis, such as *CsSWEET15*, *CsSPS* and *CsSuS*, have been identified in citrus by combining high spatiotemporal resolution transcriptome data with WGCNA (Feng GZ. et al., 2021). In peach, a total of 67 MFS family genes were systematically identified by integrating spatial metabolomics, transcriptomics and quantitative genetics. Among them, *PpERDL16–1* and *PpTST1* positively regulate sucrose accumulation, whereas *PpPMT5-1*, *PpPMT5–2* and *PpSUT4* act as negative regulators (Yang et al., 2025). Most of these candidate genes are directly involved in sugar synthesis and transport, which is consistent with the characteristics of sucrose-accumulating type fruits. In intermediate-converting type fruit trees, joint transcriptome analysis of apple revealed a significant increase in sucrose content. Further screening via WGCNA identified the transcription factor *MdWRKY75* and its downstream target genes *MdSWEET1*, *MdSS*, and *MdSPS*, which coordinately mediate sucrose transport and metabolism, and also participate in the regulation of stress response in plants (Sun et al., 2022). In pear, by integrating RNA-seq with WGCNA, co-expression modules closely associated with high sucrose accumulation have been identified, and a set of core structural genes involved in sucrose biosynthesis were screened out (Lü et al., 2020); meanwhile, through transcriptomic differential analysis, a cascade regulatory module *PuPRE6-PuMYB12-PuHDAC9-like* that modulates sucrose homeostasis was successfully elucidated (Gao et al., 2023). Genes involved in starch synthesis, sucrose transformation and carbohydrate metabolism are generally differentially enriched in these fruit tree species. This further verifies the distinctive sugar metabolic characteristics of their fruits, which are dominated by starch turnover and bidirectional sucrose conversion. In sucrose-hydrolyzing type fruit tree species, combined transcriptome sequencing and WGCNA analysis was conducted in apricot, and differentially expressed genes in sucrose metabolism pathways, key hub genes and regulatory genes were screened out (Gou et al., 2023). Transcriptomic studies in pomegranate have also successfully identified three key soluble sugar metabolism genes, including *FBA1*, SS and *SWEET16* (Ding et al., 2025). These studies lay a foundation for further dissecting the regulatory networks of hexose-accumulating fruits. However, current research remains limited to the preliminary screening of candidate genes, and a complete functional verification system has not yet been established.

In recent years, emerging omics technologies such as proteomics and epigenomics have been gradually applied to the study of sugar metabolism in fruit trees. In proteomic studies, researchers have elucidated the sugar and acid metabolism mechanism during the fruit development of Korla fragrant pear, screening a large number of differentially expressed proteins involved in sugar metabolism. These findings confirmed that dynamic changes at the protein level can coordinately regulate soluble sugar accumulation in fruits, thereby providing a protein−level theoretical basis for revealing the molecular mechanisms underlying fruit sugar and flavor formation (Qin et al., 2021). In epigenomic studies, a comparison of the epigenetic differences in apples harvested at different maturity stages revealed that epigenetic modifications are involved in regulating fruit ripening and internal quality formation. Furthermore, by affecting the transcriptional patterns of sugar metabolism-related genes, they indirectly regulate sugar accumulation and quality development in fruits (Wang et al., 2024). In another study, transcriptomic, proteomic, metabolomic, DNA methylomic, and small RNA-omic data were integrated to systematically dissect the metabolic regulatory patterns throughout the entire developmental period of pear fruit. It was revealed that DNA methylation broadly mediates fruit metabolism and sugar metabolism, thereby further refining the epigenetic regulatory framework of fruit tree fruit metabolism (Gu et al., 2024). Based on existing studies, current explorations in proteomics and epigenomics mostly focus on differential screening and mechanistic analysis of overall soluble sugar metabolism in fruits. Targeted studies specifically dedicated to the dedicated pathways of sucrose synthesis, transport, and storage remain very scarce. Therefore, at the current stage, gene discovery and mechanistic analysis of the regulatory mechanisms of sucrose in fruit trees still rely primarily on transcriptomics and metabolomics as the core mainstream research approaches. In summary, multi-omics approaches can mine candidate genes related to sugar metabolism from different molecular levels, but they can only establish correlations between genes and sugar accumulation, and cannot directly confirm their regulatory functions. Key candidate genes identified through multi-omics screening still require validation via *in vivo* functional assays such as genetic transformation and gene editing to determine their true biological functions in sucrose metabolism and sugar accumulation.

## 4 Research on molecular regulatory network of fruit sucrose accumulation

Fruit sucrose accumulation is a complex biological process synergistically regulated by three core pathways: sucrose synthesis, transmembrane transport, and vacuolar storage. Differential expression, interaction patterns, and regulatory intensity of key genes in each pathway underlie the species-specific sugar accumulation characteristics of different fruit trees. Starting from these three core pathways, this paper systematically summarizes the key regulatory elements and molecular regulatory mechanisms in the three types of fruit trees, aiming to enhance the theoretical framework of molecular regulation underlying sucrose accumulation in fruit trees. This provides a core theoretical basis for elucidating the formation mechanism of fruit sugar quality and realizing the directional improvement of fruit flavor quality.

### 4.1 Molecular regulatory mechanism of sucrose synthesis pathway

Fruit sucrose synthesis mainly relies on the coordination of SPS and SPP: SPS catalyzes the formation of sucrose-6-phosphate from fructose-6-phosphate and UDP-glucose, which is further dephosphorylated by SPP to produce sucrose (Su et al., 2021). Different from the unidirectional SPS-SPP pathway, sucrose synthase (SUS) is a key reversible enzyme involved in plant sucrose metabolism. It catalyzes the reversible conversion among sucrose, fructose and uridine diphosphate glucose (UDP-G), and maintains the dynamic balance of sucrose between source and sink organs through such reversible reactions (Sun et al., 2025). The sucrose synthesis pathway is conserved in fruits with different sucrose accumulation types. It mainly modulates the expression levels of key synthetic genes to alter the dynamic balance between sucrose synthesis efficiency and catabolism, thereby forming three distinct sugar accumulation patterns: sucrose enrichment, bidirectional transformation, and hydrolytic consumption.

In sucrose-accumulating type fruit trees, relatively few genes related to synthetic regulation have been identified in jujube. Only several key enzyme-encoding genes positively associated with sucrose synthesis have been characterized, and research on the relevant regulatory mechanisms remains insufficient (Guo et al., 2019; Li et al., 2022). Multiple precise regulatory pathways have been elucidated in citrus. The transcription factor *CitSA*R can directly bind to the promoter of *CitSPS4* to activate its transcription, thereby promoting sucrose synthesis (Liu SC. et al., 2025). Meanwhile, *CitERF36* further ensures efficient sucrose enrichment and net accumulation by activating and upregulating the sucrose transporter gene SUT2 (Pang et al., 2026).

Intermediate-converting type fruit trees are characterized by the dynamic balance of sucrose synthesis and decomposition. In apple, *MdWRKY126* can positively regulate the expression of SPS, thereby promoting sucrose biosynthesis (Zhang LH. et al., 2025). A member of the SPS gene family, *MdSPSA2.3*, is continuously highly expressed during fruit development and shows a positive correlation with sucrose content. It has been identified as one of the major-effect genes for sucrose accumulation and a core functional gene determining sucrose accumulation (Zhang LH. et al., 2023). In pear, *PbrbZIP15* activates the transcription of the downstream glucose isomerase gene *PbrXylA1*, thereby simultaneously increasing the contents of sucrose and total sugars (Jia et al., 2023).

Sucrose-hydrolyzing type fruit trees are dominated by sucrose hydrolysis metabolism. In grape, sucrose synthase (SS) mainly functions in sucrose degradation. Studies have shown that the sucrose synthase gene *VvSS3* interacts with the protein kinase *VvSnRK1β* to form a complex, which coordinately responds to ABA signals, accelerates sucrose degradation, and promotes massive accumulation of hexoses (Hong et al., 2023).

In summary, the three types of fruit trees all take SPS and SUS/SS as the core functional elements for sucrose synthesis, yet their regulatory patterns are completely distinct: the sucrose-accumulating type relies on high expression of SPS and the synthetic-biased activity of SUS/SS to achieve directional enrichment of sucrose; in the intermediate-converting type, SUS/SS coordinates and balances synthesis and hydrolysis pathways. Part of the synthetic products are consumed by the hydrolysis pathway, forming a metabolic feature with coexistence of sucrose and hexose; the sucrose-hydrolyzing type is dominated by the degradative activity of SUS/SS, which cooperates with invertase to continuously degrade sucrose, ultimately forming a sugar composition pattern dominated by hexose accumulation.

### 4.2 Molecular regulatory mechanism of sucrose transport pathway

Sucrose transport acts as a central hub pathway linking sucrose synthesis and vacuolar storage, which is dominated by transporter families including sugar transporters SWEET, proton-sucrose symporters SUT, and tonoplast sugar transporters TST. Among them, SWEET and SUT constitute the two major transporter families. SWEETs mediate the passive efflux of sucrose into the apoplast, while SUTs are responsible for actively loading sucrose into the phloem or sink cells (Chen et al., 2024). The two families coordinately regulate the directional transport of sucrose.

There is significant functional differentiation and species specificity among members of the SWEET and SUT transporter families in different types of fruit trees. In sucrose-accumulating type fruit trees including jujube and citrus, a total of 19 *ZjSWEET* genes have been identified at the genome-wide level in jujube, which are inferred to participate in sugar accumulation and underpin the high sucrose accumulation characteristic of jujube fruit (Yang et al., 2023). In citrus, *CitSWEET15* mediates the apoplastic transmembrane transport of sucrose to facilitate sucrose enrichment during fruit ripening. Meanwhile, *CitSWEET6* is capable of transporting fructose and is positively regulated by *CitZAT5*, which modulates the proportion of hexose in fruit (Fang et al., 2023; Zheng et al., 2023). Studies on citrus have shown that the expression of the sucrose transporter *CsSUT2* is directly regulated by the transcription complex *CsbHLH122/CsMYBS3*. This complex binds to its promoter and activates transcription, thereby significantly enhancing the efficiency of sugar unloading and accumulation during fruit ripening; its function has been verified by ^14^C labeling experiments (Zhai et al., 2025).

In intermediate-converting type apple, *MdSWEET9b* is hierarchically regulated by *MdWRKY9* combined with ABA signaling pathway factors and functions in the specific transport of sucrose. Meanwhile, *MdWRKY9* interacts with ABA signal transduction factors *MdbZIP23* and *MdbZIP46*, enhancing the regulatory effect on the expression of *MdSWEET9b* and thereby affecting sugar accumulation (Zhang SH. et al., 2023). In addition, *MdSWEET23* cloned from the ‘Hanfu’ apple is localized on the plasma membrane and highly expressed in the vascular bundles of sepals and carpels, with sucrose transport activity. Overexpression of MdSWEET23 increases the content of soluble sugars, whereas its silencing significantly reduces the levels of sucrose and sorbitol. These results indicate that *MdSWEET23* positively regulates the accumulation of soluble sugars, sucrose and sorbitol in fruits (Nie et al., 2023). Meanwhile, the type II sucrose transporter *MdSUT2.1* in apple is positively correlated with sucrose accumulation. It mainly functions during the sucrose loading stage and ensures the long-distance transport of sucrose toward fruits (Zhang B. et al., 2023).

In sucrose-hydrolyzing type fruit trees, *PaSWEET1* is responsible for sucrose unloading at the source end, providing substrates for downstream sucrose metabolism (Jiang et al., 2025). In strawberry, *FaSWEET9a* has also been confirmed to possess sucrose transport activity. Its expression is enhanced when the transcription factor *FaDOF2* directly binds to its promoter. Overexpression of *FaSWEET9a* promotes sucrose accumulation in strawberry fruits and also affects plant growth and development (Xu et al., 2025). The above studies indicate that the SWEET family plays a pivotal role in sucrose unloading and accumulation in the fruits of various fruit tree species.

In conclusion, the fundamental functional division of SWEET and SUT transporters in fruit trees is evolutionarily conserved across species: SWEETs mainly mediate passive sucrose efflux and apoplastic unloading, while SUTs are responsible for active sucrose loading and long-distance transport. Moreover, both types of transporter genes are precisely regulated by upstream transcription factors. However, there are marked differences in regulatory intensity and functional emphasis of the transport systems among the three types of fruit trees: the overall activity of transporters in sucrose-accumulating type fruit trees is higher, focusing on efficient sucrose unloading and enrichment; the transport system of intermediate-converting type fruit trees features balanced functions, adapting to sucrose turnover and bidirectional metabolism in fruits; transporters in sucrose-hydrolyzing type fruit trees are mainly responsible for carbon source input, providing substrates for subsequent sucrose hydrolysis and hexose accumulation.

### 4.3 Molecular regulatory mechanism of sucrose storage pathway

The vacuole is the central site for sucrose storage in fruits. Tonoplast transporter families such as TST and VPS mediate the transmembrane transport, vacuolar enrichment, and compartmental sequestration of sucrose while maintaining cellular sucrose homeostasis, serving as the terminal core mechanism that determines the sucrose storage capacity and sugar differentiation phenotypes of different fruit tree species. In sucrose-accumulating type citrus, an elaborate post-translational modification regulatory pathway exists. Calcium signaling phosphorylates the tonoplast transporter *CsTST2* via the *CBL1/CIPK23* kinase complex, markedly enhancing its sucrose transport activity. This efficiently drives sucrose enrichment from the cytoplasm into vacuoles and strengthens fruit sucrose storage capacity at the protein modification level, acting as a key mechanism for efficient sugar storage in sucrose-accumulating type fruit trees (Li MD. et al., 2025). In peach, the subgroup I bZIP transcription factor *PpbZIP18* simultaneously activates the expression of the sucrose synthase gene *PpSuSy1* and the sugar transporter gene *PpST1*. *PpSuSy1* catalyzes the decomposition of sucrose to provide substrates for sugar accumulation, while *PpST*1 mediates sucrose transport into fruit cells, jointly promoting sugar accumulation (Zhang X. et al., 2025). In intermediate-converting type apple, a closed-loop regulatory system dependent on endogenous sugar signals has formed. The vacuolar efflux protein *MdERDL6* modulates cytoplasmic glucose homeostasis, which further activates the *SnRK2.3-AREB1* kinase-transcription factor cascade pathway and upregulates the expression of vacuolar influx proteins *MdTST1/2*. Through a dynamic closed loop of “efflux sensing-influx enrichment”, it balances sucrose turnover and storage, adapting to the bidirectional carbon metabolism turnover characteristics of intermediate-converting type fruit trees (Lingcheng et al., 2023). In sucrose-hydrolyzing type fruit trees, transcription factors *FvNAC073* and *FvCMB1L* in strawberry competitively bind to the promoters, antagonistically regulating the expression of the sucrose synthesis gene *FvSPS1* and decomposition gene *FvSUS2*. This dynamically balances the efficiency of sucrose synthesis and hydrolysis, stabilizes the net sucrose accumulation level in fruits, and provides sufficient substrates for vacuolar storage of sucrose (Xiao et al., 2025). On this basis, tonoplast transporters of the MFS superfamily such as TST/TMT and *ERDL6* are responsible for sucrose transmembrane transport across the vacuolar membrane. Meanwhile, metabolic enzymes maintain the sugar concentration gradient between the cytoplasm and vacuole, which provides the driving force for sucrose transport (Zhu et al., 2025). The three types of fruit trees exhibit significant differences in the regulatory patterns of the sucrose storage pathway: Sucrose-accumulating type fruit trees achieve efficient sucrose sequestration by enhancing the activity of vacuolar transporters; intermediate-converting type fruit trees balance sucrose storage and turnover through metabolic feedback; Sucrose-hydrolyzing type fruit trees have weakened vacuolar sugar storage capacity, and sucrose is more readily hydrolyzed and converted into hexose.

**Figure 2:**
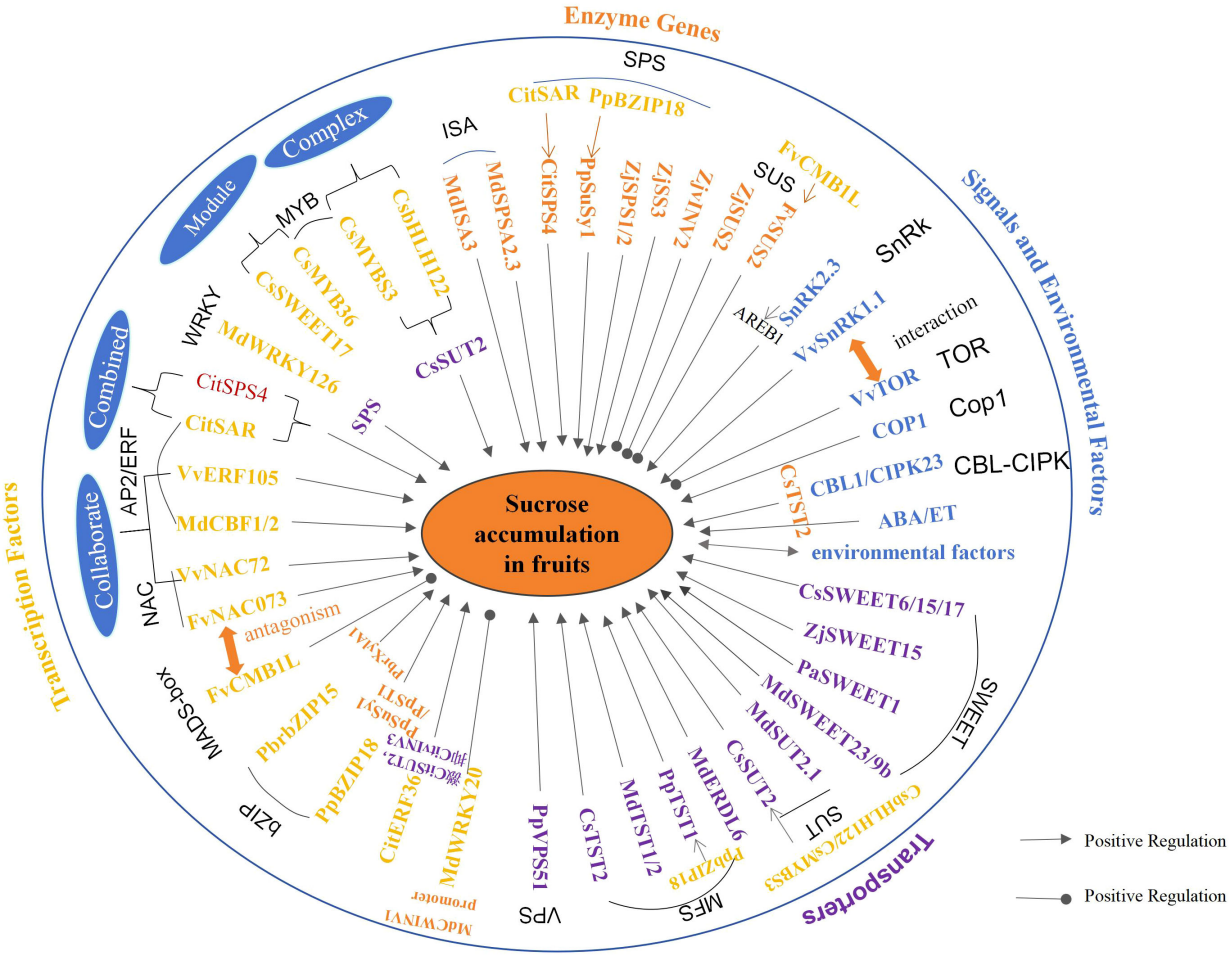
Regulatory network of sucrose metabolism in fruit tree fruits. This image illustrates the molecular regulatory network of sucrose metabolism in fruits of major fruit tree species including apple, citrus, peach, pear, strawberry, grape and jujube. Taking sucrose accumulation in fruit as the final phenotypic outcome, it integrates functional components such as enzyme genes, transcription factors, transporter proteins, as well as signaling and environmental factors, and visually demonstrates how each regulatory element modulates sucrose accumulation in fruit.

In summary, sucrose accumulation in fruit tree fruits relies on three core pathways: sucrose synthesis, transport and storage, forming a sophisticated multi-level and multi-factor synergistic molecular regulatory network (**Figure 2**). Within the overall regulatory system, the interaction of transcription factors constitutes the core of upper-level regulation, which precisely modulates the transcriptional levels of key genes in related pathways; metabolic enzymes such as SPS and SUS, together with transporters including SUT, SWEET and TST, act as core functional components to execute the entire processes of sucrose synthesis, intercellular transport and vacuolar storage; post-translational protein modification and endogenous metabolic feedback serve as fine regulatory switches, coordinating upstream and downstream signaling pathways to adapt to the process of fruit development. Fruit trees with different sucrose accumulation types exhibit distinct mechanistic differentiation: sucrose-accumulating type fruit trees achieve sucrose enrichment by enhancing the capacity of sucrose synthesis as well as vacuolar transport and storage, while weakening hydrolytic metabolism; intermediate-converting type fruit trees maintain dynamic balance among the three major pathways to complete the bidirectional carbon turnover of sucrose hydrolysis and regeneration; Sucrose-hydrolyzing type fruit trees tend to activate the sucrose hydrolysis pathway to promote the accumulation of hexose. The differential and coordinated operation of this multi-level pathway ultimately determines sucrose metabolic accumulation in flesh cells, resulting in distinct sugar accumulation phenotypes among fruit tree species (**Table 2**).

**Table 2:** Regulatory genes of sucrose and their functions in fruit tree fruits.

Type	Name	Plant species	Function in sucrose metabolism	Regulate the direction of sucrose	References
Enzyme Gene	*MdISA3*	Apple	Isoamylase gene; overexpression of dominant haplotype significantly enhances sucrose accumulation	Positive	(Wang, 2022)Wang, L et al.
	*PpTST1*	Peach	Tonoplast monosaccharide transporter; non-synonymous mutation affects sucrose content	Positive	(Shi, 2021)Shi, P et al.
	*ZjSS3*	Jujube	Sucrose synthase gene; positively correlated with sucrose accumulation	Positive	(Wang et al., 2022)Wang, Y et al.
	*ZjSPS1*	Jujube	Sucrose phosphate synthase gene; positively correlated with sucrose accumulation	Positive	(Wang et al., 2022)Wang, Y et al.
	*ZjSPS2*	Jujube	Sucrose phosphate synthase gene; positively correlated with sucrose accumulation	Positive	(Wang et al., 2022)Wang, Y et al.
	*ZjvINV2*	Jujube	Vacuolar acid invertase gene; negatively correlated with sucrose accumulation	Negative	(Wang et al., 2022)Wang, Y et al.
	*ZjSUS2*	Jujube	Sucrose synthase; high expression in cultivated jujube promotes sucrose accumulation	Negative	(Zhang Z. et al., 2025)Zhang Z et al.
	*CsSPS1-4*	Citrus	Sucrose phosphate synthase gene family; positively correlated with sugar accumulation	Positive	(Lu W. et al., 2024)Lu W et al.
	*MdCWINV1*	Apple	Cell wall invertase; promoter variation affects fructose accumulation	Negative	(Wang et al., 2026)Wang Z et al.
	*MdSPSA2. 3*	Apple	Sucrose phosphate synthase gene; positively correlated with sucrose content	Positive	(Zhang LH. et al., 2023)Li-hua Z et al.
	*VvSS3*	Grape	Sucrose synthase interacts with *VvSnRK1β* to promote the decomposition of sucrose	Negative	(Hong et al., 2023)Hong, P et al.
	*FvSPS1*	Strawberry	Sucrose phosphate synthase gene; activated by *FvNAC073*	Positive	(Xiao et al., 2025)Xiao K et al.
	*FvSUS2*	Strawberry	Sucrose synthase gene; activated by *FvCMB1L* and involved in sucrose degradation	Negative	(Xiao et al., 2025)Xiao K et al.
	*PpSuSy1*	Peach	Sucrose synthase gene; activated by *PpbZIP18*	Positive	(Zhang X. et al., 2025)Zhang X et al.
Transcription Factor	*CitSAR*	Citrus	Activates *CitSPS4* to promote sucrose synthesis	Positive	(Liu SC. et al., 2025)Liu S et al.
	*CitERF36*	Citrus	Activates *CitSUT2* and inhibits *CitvINV3*, promoting sucrose transport and suppressing sucrose degradation	Positive	(Pang et al., 2026)Pang Y et al.
	*CsMYBS3*	Citrus	Interacts with *CsbHLH122* to activate the transcription of *CsSUT2*	Positive	(Zhai et al., 2025)Zhai X et al.
	*CsbHLH122*	Citrus	Interacts with *CsMYBS3* to activate the transcription of *CsSUT2*	Positive	(Zhai et al., 2025)Zhai X et al.
	*MdWRKY20*	Apple	Binds to the promoter of *MdCWINV1* and negatively regulates fructose accumulation	Negative	(Wang et al., 2026)Wang Z et al.
	*PbrbZIP15*	Pear	Activates *PbrXylA1* to promote fructose and sucrose accumulation	Positive	(Jia et al., 2023)Jia, L et al.
	*VvERF105*	Grape	Cooperates with *VvNAC72* to temporally regulate hexose transporters	Negative	(Lu et al., 2024a)Lu L et al.
	*VvNAC72*	Grape	In synergy with *VvERF105*, it decomposes sucrose and promotes the accumulation of hexose	Negative	(Lu et al., 2024a)Lu L et al.
	*MdWRKY126*	Apple	Upregulates SPS expression to promote sucrose accumulation	Positive	(Zhang LH. et al., 2025)Zhang L et al.
	*FvNAC073*	Strawberry	Activates *FvSPS1* and inhibits *FvSUS2* to promote sucrose accumulation	Positive	(Xiao et al., 2025)Xiao K et al.
	*FvCMB1L*	Strawberry	Inhibits *FvSPS1* and activates *FvSUS2*, antagonizing *FvNAC073*	Negative	(Xiao et al., 2025)Xiao K et al.
	*PpbZIP18*	Peach	Activates *PpSuSy1* and *PpST1* to promote sucrose synthesis and transport	Positive	(Zhang X. et al., 2025)Zhang X et al.
	*MdCBF1/2*	Apple	Responds to low temperature, activates *MdTST1/2*, and promotes vacuolar sucrose accumulation	Positive	(Li et al., 2024)Li B et al.
Transporter Protein	*ZjSWEET15*	Jujube	SWEET family; mediates sucrose unloading and promotes sucrose accumulation	Positive	(Zhang Z. et al., 2025)Zhang Z et al.
	*CsSUT2*	Citrus	SUT family; activated by *CsbHLH122*/*CsMYBS3*, mediates sucrose transport	Positive	(Zhai et al., 2025)Zhai X et al.
	*PaSWEET1*	Apricot	SWEET family; mediates sucrose unloading at the source end	Positive	(Jiang et al., 2025)Jiang F et al.
	*CitSWEET15*	Citrus	SWEET family; plasma membrane-localized, upregulated during fruit ripening, mediates transmembrane sucrose transport	Positive	(Zheng et al., 2023)Zheng, Q et al.
	*CitSWEET6*	Citrus	Fructose transporter; positively regulated by *CitZAT5*	Positive	(Fang et al., 2023)Fang, H et al.
	*MdSWEET9b*	Apple	SWEET family; regulated by *MdWRKY9* and ABA, specifically transports sucrose	Positive	(Zhang SH. et al., 2023)Zhang, S et al.
	*MdSWEET23*	Apple	SWEET family; plasma membrane-localized, overexpression increases soluble sugar content	Positive	(Nie et al., 2023)Nie, P et al.
	*MdSUT2. 1*	Apple	SUT family; involved in phloem loading and long-distance sucrose transport	Positive	(Zhang B. et al., 2023)Zhang, B et al.
	*FaSWEET9a*	Strawberry	SWEET family; activated by *FaDOF2* to promote sucrose accumulation	Positive	(Xu et al., 2025)Xu Y et al.
	*CsTST2*	Citrus	Tonoplast sugar transporter; phosphorylated and activated by *CBL1*/*CIPK23*	Positive	(Li MD. et al., 2025)Li M et al.
	*MdTST1/2*	Apple	Tonoplast sugar influx protein; activated by *AREB1*	Positive	(Zhu et al., 2025)Zhu L et al.
	*MdERDL6*	Apple	Vacuolar glucose efflux protein; maintains cytoplasmic sugar concentration	Positive	(Zhu et al., 2025)Zhu L et al.
	*PpTST1*	Peach	Sugar transporter; activated by *PpbZIP18* to facilitate sucrose transport	^Positive^	(Zhang X. et al., 2025)Zhang X et al.
Signaling and Environmental Factor	*COP1*	Strawberry	E3 ubiquitin ligase; inhibits sugar synthesis-related factors	Negative	(Bi et al., 2025)Bi X et al.
	*VvSnRK1β*	Grape	It interacts with *VvSS3* to form a complex, responds to ABA signals, and accelerates sucrose decomposition	Negative	(Hong et al., 2023)Hong, P H et al.
	*FaDOF2*	Strawberry	Transcription factor; activates *FaSWEET9a*	Positive	(Xu et al., 2025)Xu Y et al.
	*CBL1/CIPK23*	Citrus	Calcium signaling kinase module; phosphorylates *CsTST2*	Positive	(Li MD. et al., 2025)Li M et al.
	*SnRK2. 3*	Apple	Kinase; activates *AREB1* via phosphorylation	Positive	(Zhu et al., 2025)Zhu L et al.
	*AREB1*	Apple	Transcription factor; activated by *SnRK2. 3* to induce *MdTST1/2* expression	Positive	(Zhu et al., 2025)Zhu L et al.
	*VvTOR*	Grape	Kinase; interacts with *VvSnRK1. 1* to regulate sugar metabolism	Positive	(Zhao and Wang, 2022)Zhao, Y et al.
	*VvSnRK1. 1*	Grape	Kinase; interacts with *VvTOR* and participates in the regulation of sugar metabolism	Positive	(Zhao and Wang, 2022)Zhao, Y et al.
	ABA	Peach	Hormone; induces *PpbZIP18* to promote sugar accumulation	Positive	(Zhang X. et al., 2025)Zhang X et al.
	ET	Apple	Hormone; promotes the expression of *MdSPS*/*MdSUSY*	Positive	(Si et al., 2026)Si Y et al.
	low temperature	Apple	Environmental factor; activates *MdCBF1/2* and upregulates *MdTST1/2*	Positive	(Li et al., 2024)Li B et al.
	light	Grape	Environmental factor; regulates the expression of sucrose transport genes	Bidirectional	(Liu et al., 2021)Liu, S et al.
	Drought	Citrus	Environmental factor; promotes sucrose synthesis through ABA signaling pathway	Positive	(Zeng et al., 2025)Zeng Y et al.

This table systematically summarizes the gene names, specific functions and regulatory patterns of key components involved in the core “synthesis-transport-storage” pathways and their regulatory networks during sucrose accumulation in fruits of various fruit trees, which collectively demonstrates the research progress in gene mining and functional characterization in this field.

### 4.4 Upstream regulatory mechanism mediated by hormones and environmental factors

In addition to hierarchical regulation by endogenous genes, the synthesis, transport and storage pathways of fruit sucrose are also universally modulated upstream by plant hormones and external environmental signals, which constitute the core upstream regulatory module of the sucrose metabolic network. Endogenous hormones such as abscisic acid, ethylene and gibberellin can target and regulate sucrose metabolism-related genes and transcription factors through signal transduction pathways, dominating the process of sucrose metabolism and accumulation in fruits at the endogenous level. Environmental factors such as light, temperature and cultivation methods can alter the gene expression and enzyme activity of sugar metabolic pathways, thereby achieving adaptive remodeling of the sugar metabolism network. Endogenous hormones interact with external environmental signals to coordinately regulate the three core pathways of sucrose synthesis, transport and storage, collectively determining the sugar accumulation efficiency and flavor quality of fruit tree fruits. Sucrose itself acts as a sensor for perceiving endogenous sugar levels. It regulates growth, development and metabolism through a series of signal transduction processes to maintain intracellular homeostasis (Feng YL. et al., 2021).

Abscisic acid (ABA) and ethylene, as the core endogenous hormones regulating fruit ripening and sugar accumulation, are extensively involved in the whole processes of fruit sucrose synthesis, transport and storage by mediating multi-level signaling pathways and regulating the expression of transcription factors and functional genes. At the level of sucrose synthesis, ABA in peach can induce the expression of subgroup I bZIP transcription factor *PpbZIP18*, activate the downstream sucrose synthesis gene PpSuSy1, and enhance the sucrose synthesis capacity of fruits (Zhang X. et al., 2025). Under drought conditions, citrus stabilizes the *WRKY41* protein relying on the ABA signaling pathway, and forms a dual regulatory pathway in coordination with *WRKY23*, which jointly activates the transcription of SPS4 and significantly promotes sucrose synthesis (Zeng et al., 2025). Ethylene also positively regulates sucrose anabolism. After ethylene production is inhibited in apple fruits, the expression of key sucrose synthesis genes *MdSPS* and *MdSUSY* is significantly downregulated, confirming that ethylene signaling is closely associated with the activity of sucrose synthetase (Si et al., 2026). At the level of sucrose transport and storage, ABA serves as the dominant regulatory hormone. In grape, ABA remodels the expression patterns of transcription factors such as MYB, ERF, and bZIP, upregulates sugar transport genes including *VvSWEET15* and *VvHT1*, and meanwhile inhibits genes related to sucrose hydrolysis, thereby promoting sugar accumulation in fruits (Mu et al., 2025). In citrus, ABA couples with calcium signaling to activate the vacuolar transporter gene TST2 via the *SRK2A/CIPK6-ABI5* signaling module, mediating the transmembrane transport and storage of sucrose in vacuoles (Mao et al., 2025). In peach, ABA-induced *PpbZIP18* not only regulates sucrose synthesis but also activates the sugar transport gene *PpST1*, simultaneously enhancing the efficiency of sucrose transport (Zhang X. et al., 2025). In addition, sucrose can act as a signaling molecule in reverse to feedback regulate the response intensity of ABA and ethylene signals, forming an interactive regulatory network with hormone pathways and coordinately modulating the processes of fruit ripening and sugar accumulation (Durán-Soria et al., 2020).

External environmental factors such as light, temperature and water stress, as well as intracellular endogenous energy-sensing signals, can adaptively regulate sucrose metabolism and homeostatic accumulation in fruits by altering the transcriptional level, protein activity and pathway response patterns of sugar metabolism-related genes. Light is a crucial external regulatory factor. Different light quality treatments during the veraison stage of grape can significantly regulate the transcriptional expression of sucrose transport genes, and further alter sucrose accumulation efficiency and flavor quality of fruits (Liu et al., 2021). Drought stress can activate the ABA-dependent signaling pathway in fruit trees, regulate the functions of SWEET family transporter proteins through phosphorylation modification, reshape the sucrose source-sink allocation pattern, and prioritize the stress survival metabolism of plants (Chen et al., 2021). Low temperature stress mainly regulates the vacuolar storage process of sucrose in fruits. In apple, low temperature can activate the core transcription factor CBF, which specifically binds to and upregulates the expression of vacuolar transporter genes *MdTST1/2*, promoting the compartmentalized sequestration of sucrose in vacuoles and achieving net sucrose accumulation in fruits (Li et al., 2024). Meanwhile, the intracellular endogenous energy-sensing pathway acts as a core hub regulating sucrose metabolism. TOR and SnRK1 proteins form an antagonistic regulatory system. In grape, *VvTOR* and *VvSnRK1.1* modulate sucrose accumulation efficiency during fruit ripening through protein interaction (Zhao and Wang, 2022). In addition, the TOR pathway serves as the core hub balancing plant carbon and nitrogen metabolism. It coordinately regulates sugar metabolism and nitrogen assimilation processes by phosphorylating downstream target genes, profoundly affecting sucrose accumulation and quality formation in the later stage of fruit development (Artins et al., 2024).

In summary, endogenous hormones, external environmental factors and cellular energy signals act as upstream regulatory signals that precisely target the three core pathways of sucrose synthesis, transport and storage. They dynamically regulates the functional activity of sugar metabolic pathways and the source-sink carbon allocation pattern through transcriptional regulation, protein modification and signal interaction, and mediates the formation of differential sucrose accumulation phenotypes of fruit trees at different developmental stages and under stress conditions. This serves as the key upstream regulatory mechanism underlying the diversity of sugar quality in fruit tree fruits.

## 5 Future perspectives

This review systematically summarizes and constructs the molecular regulatory system of the three major pathways—synthesis, transport, and storage—for sucrose accumulation in fruit tree fruits. It clarifies the differential metabolic characteristics of three types of fruit tree fruits, namely the sucrose-accumulating, intermediate-converting, and sucrose-hydrolyzing types, and summarizes the key structural genes, transporter genes, and upstream regulatory elements involved in the corresponding pathways. Current studies have identified numerous candidate genes and metabolic modules regulating sugar accumulation, such as MdISA3, PpTST1, and MdCWINV1, through QTL mapping and multi-omics analysis, laying a foundation for understanding the mechanism of sucrose accumulation. However, several shortcomings and bottlenecks remain in current research on this topic. Most sugar-related QTLs span broad mapping intervals, making it difficult to pinpoint functional genes precisely. The analysis of multi-gene interactions and pathway synergistic networks in sucrose metabolism remains incomplete. The stable genetic transformation system of woody fruit trees is still immature; relying solely on transient transformation can only preliminarily verify the functions of metabolic genes, which greatly restricts the causal validation and in-depth mechanistic exploration of gene functions. Meanwhile, research on the regulatory mechanisms of post-translational modifications (e.g., phosphorylation and ubiquitination) governing the activity of vacuolar storage proteins remains scarce. In addition, the crosstalk regulatory network among environmental cues, hormones, and sugar signaling has not been fully clarified, which hampers the systematic elucidation of the multi-layered regulatory mechanism of sucrose accumulation in fruits.

In view of the above research deficiencies, future studies on fruit sugar quality should build on the existing “synthesis–transport–storage” regulatory framework and further advance in four key directions: multi-layered regulatory dissection, environment–gene interaction, multi−trait pyramiding, and precision molecular breeding. First, the genetic transformation system of woody fruit trees should be optimized to establish a stable, efficient, and universal platform for gene functional verification. This will help overcome the limitations of transient transformation assays, enable the functional characterization of key sugar−regulatory genes, and shift the research focus from correlation analysis to causal mechanism elucidation. Second, sucrose accumulation is a typical complex quantitative trait controlled by multiple genes. Most existing studies are limited to single−gene or single−pathway analyses. Future research should integrate multi−omics data (including transcriptomics, metabolomics, and epigenomics) with precise environmental monitoring via the Internet of Things and big data analysis to quantify the regulatory effects of environmental factors (e.g., light, temperature, and water) on the sugar metabolic network and to construct an environment−responsive collaborative regulatory network model for sucrose accumulation. Moreover, artificial intelligence and machine learning algorithms can be employed to accurately screen core transcription factors, interacting proteins, and key functional genes, thereby improving the mining accuracy of regulatory modules in sugar metabolism. Third, further efforts should be made to dissect the regulatory mechanisms of post−translational modifications on the activity of key proteins involved in sucrose transport and storage, to elucidate the fine regulatory mechanism of sugar homeostasis mediated by protein modifications, and to refine the molecular regulatory theory at the level of sucrose storage. Fourth, the crosstalk between hormone signals and sugar signals should be systematically dissected, the functional differentiation patterns of transporter family members (e.g., SUT and SWEET) clarified, and the coupling between sugar accumulation and other fruit quality traits (e.g., acidity and color) explored to achieve the synergistic regulation of multiple quality traits. At the breeding application level, it is necessary to accelerate the breeding deployment of superior alleles such as MdCWINV1 and PpST1, combine genomic selection with molecular marker−assisted breeding technologies, break the genetic linkage among traits such as quality and stress resistance, and achieve the precise improvement of multiple traits (high sugar content, high quality, and stress resistance) in fruits. In summary, integrating basic mechanistic research with breeding−application development can break through the current technical bottlenecks in the study of sucrose accumulation in fruit trees and improve the molecular regulatory system that governs fruit sugar quality formation. Ultimately, this provides a solid theoretical basis and technical support for the precise design breeding of fruit trees, the cultivation of high−quality fruits, and the improvement of industrial quality and efficiency.
